# Development of a multiplex polymerase chain reaction assay for simultaneous identification of human enterovirus 71 and coxsackievirus A16

**DOI:** 10.1016/j.jviromet.2010.09.017

**Published:** 2010-12

**Authors:** Nguyen Thi Thanh Thao, Nguyen Thi Kim Ngoc, Phan Văn Tú, Trần Thi Thúy, Mary Jane Cardosa, Peter Charles McMinn, Patchara Phuektes

**Affiliations:** a Pasteur Institute of Ho Chi Minh City, 167 Pasteur Street, District 3, Ho Chi Minh City, Viet Nam; b Children's Hospital Number 2, 14 Ly Tu Trong St, W. Ben Nghe, District 1, Ho Chi Minh City, Viet Nam; c Institute for Health and Community Medicine, Universiti Malaysia Sarawak, Sarawak, Malaysia; d Infectious Diseases and Immunology, Central Clinical School, The University of Sydney, New South Wales, Australia

**Keywords:** Enterovirus, Multiplex RT-PCR, Hand, foot and mouth disease

## Abstract

Human enterovirus 71 (HEV71) and coxsackievirus A16 (CVA16) are two major aetiological agents of hand, foot and mouth disease (HFMD) in children. Recently there have been several large outbreaks of HFMD in Vietnam and the Asia-Pacific region. In this study, a multiplex RT-PCR assay was developed in order to detect simultaneously HEV71, CVA16 and other human enteroviruses. Enterovirus detection was performed with a mixture of three pairs of oligonucleotide primers: one pair of published primers for amplifying all known enterovirus genomes and two new primer pairs specific for detection of the VP1 genes of HEV71 and CVA16. Enterovirus isolates, CVA16 and HEV71 strains identified previously from patients with HFMD were examined to evaluate the sensitivity and specificity of the multiplex RT-PCR assay. The assay was then applied to the direct detection of these viruses in clinical specimens obtained from HFMD cases identified at Children's Hospital Number 2, Ho Chi Minh City, Vietnam. The multiplex RT-PCR assay showed 100% specificity in screening for enteroviruses and in identifying HEV71 and CVA16. Similar results were obtained when using the multiplex RT-PCR assay to screen for enteroviruses and to identify HEV71 and CVA16 in clinical specimens obtained from HFMD cases identified at the hospital. This multiplex RT-PCR assay is a rapid, sensitive and specific assay for the diagnosis of HEV71 or CVA16 infection in cases of HFMD and is also potentially useful for molecular epidemiological investigations.

## 1 Introduction

Hand, foot and mouth disease (HFMD) is a common childhood exanthem, characterised by a brief febrile illness and vesicular lesions on the hands, feet, mouth and buttocks (Grist et al., 1978). Coxsackievirus A16 (CVA16) and human enterovirus 71 (HEV71), members of the human enterovirus A (HEV-A) species, are the two major causative agents of HFMD (McMinn, 2002; Pallansch and Roos, 2007). Other members of HEV-A, including CVA2, CVA4, CVA5, CVA6, CVA8 and CVA10, and also members of HEV-B species, including echovirus (ECHO) types 1, 4, 6, 7, 19 and 25, CVA9, CVB3, CVB4 and CVB5, have also been associated with outbreaks or sporadic cases of HFMD (Abubakar et al., 1999; Ang et al., 2009; Chan et al., 2003; Chua et al., 2001; Hooi et al., 2002; Moreira et al., 1995; Russo et al., 2006; Zhu et al., 2007). Although HFMD caused by infection with these enteroviruses is clinically indistinguishable, infection with HEV71 is commonly associated with acute neurological disease, including aseptic meningitis, brainstem encephalitis, and acute flaccid paralysis (AFP), which may lead to permanent paralysis or death (McMinn, 2002). By contrast, HFMD due to CVA16 and other enteroviruses rarely results in neurological complications (Pallansch and Roos, 2007).

Over the past decade, there has been an increase in the prevalence of HFMD and acute neurological disease caused by HEV71 infection in the Asia-Pacific region (Fujimoto et al., 2002; Huang et al., 1999; Lin et al., 2003; McMinn, 2002; Podin et al., 2006; Tu et al., 2007). In southern Vietnam, HFMD is recognised frequently in children and is considered a good clinical indicator of outbreaks of HEV71-induced encephalitis. In 2005, 411 enteroviruses were isolated from HFMD cases in southern Vietnam. Of these, 173 (42%) were identified as HEV71 and 214 (52%) as CVA16 (Tu et al., 2007). Nearly one-third of the HEV71-associated HFMD cases were found to be complicated by acute neurological disease (Tu et al., 2007). With the recent increase in prevalence of HEV71 infection in southern Vietnam, it has become necessary to develop a rapid, sensitive, specific and cost-effective assay to distinguish HFMD caused by HEV71 or CVA16.

Until recently, virus isolation from faecal and throat swab samples was the primary method for the diagnosis of enterovirus infection. Serotype identification of isolated virus was achieved by neutralisation with serotype-specific antisera. More recently, reverse transcriptase–polymerase chain reaction (RT-PCR) amplification and nucleotide sequencing of the VP1 gene has replaced neutralisation as the primary method for enterovirus “serotype” identification (Brown et al., 2000; Leitch et al., 2009; Oberste et al., 2003; Perera et al., 2004). Multiplex RT-PCR assays, in which several RNA viral targets are amplified in a single reaction, allow simultaneous screening for multiple pathogens and have proven to be effective at improving the rapidity of viral diagnosis (Naze et al., 2009; Wang et al., 2009). In this study, a multiplex RT-PCR assay was developed as a rapid and cost effective tool to screen clinical specimens from HFMD cases for the presence of human enteroviruses and to identify and distinguish HEV71 and CVA16.

## 2 Materials and methods

### 2.1 HFMD case identification

One hundred and eight children admitted to the Children's Hospital Number 2, Ho Chi Minh City, Vietnam, from April to August 2009, inclusive, with a diagnosis of HFMD were enrolled in the study. HFMD was defined as a febrile illness (>37.5 °C), accompanied by a papulovesicular rash of the oral mucosa, limb extremities and/or buttocks. Each enrolled child had throat swab and/or stool specimens collected, which were transported immediately at 4 °C to the Pasteur Institute of Ho Chi Minh City (PI-HCMC), Vietnam. A total of 108 stool specimens and 104 throat swabs were collected from each of the 108 enrolled cases of HFMD for development and validation of the multiplex RT-PCR assay.

### 2.2 Virus isolation

Virus isolation was undertaken in cell culture using human rhabdomyosarcoma (RD) (ATCC CCL136) cells. Each specimen underwent at least two passages in RD cells before being reported as negative. Samples demonstrating viral cytopathic effect (CPE) were identified as HEV71 or CVA16 by HEV71-specific RT-PCR (Perera et al., 2004) or by neutralisation assay using CVA16-specific antisera (Hagiwara et al., 1978), respectively. Enteroviruses that were not identified as HEV71 or CVA16 were further identified by neutralisation with specific antisera (National Institute of Public Health and the Environment, Bilthoven, The Netherlands).

### 2.3 RNA extraction from cell culture supernatant

Viral RNA was extracted from 140 μL of cell culture supernatant using spin columns (QIAGEN, Melbourne, Australia) following the manufacturer's instructions.

### 2.4 RNA extraction from clinical specimens

Faecal specimens were initially subjected to a 10% chloroform extraction in phosphate buffered saline (PBS) containing MgCl_2_ and CaCl_2_, pH 7.4, for 10 min and then shaken vigorously for 20 min in a mechanical shaker, followed by centrifugation at 1500 × *g* for 20 min. Throat swab specimens were vortexed and the cotton stick removed before centrifuging for 15 min at 10,000 × *g*. Viral RNA was extracted from 140 μL of the faecal extracts and throat swab supernatants using spin columns (QIAGEN, Melbourne, Australia) following the manufacturer's instructions.

### 2.5 Pan-enterovirus and HEV71-specific RT-PCR assays

Pan-enterovirus and HEV71-specific RT-PCR assays were performed on RNA extracts from clinical specimens as previously described (Tu et al., 2007).

### 2.6 Oligonucleotide primers used in a multiplex RT-PCR assay

Oligonucleotide primers F1 and R1 (Zoll et al., 1992), targeting the highly conserved 5′ untranslated region (5′UTR), were used to amplify all known enterovirus genomes. Two sets of primer pairs specific for the VP1 genes of HEV71 and CVA16 were designed based on nucleotide sequences obtained from GenBank. The primer sequences are shown in Table 1.

### 2.7 Viral cDNA synthesis

Viral cDNA was prepared in a 10 μL reaction mixture containing 6 μL of RNA extracted from infected cells or clinical specimens, 0.5 μg of random primers (Promega, Sydney, Australia), 0.5 mM dNTPs, 100 U M-MLV RT (Promega, Sydney, Australia), and M-MLV RT buffer (Promega, Sydney, Australia). cDNA synthesis was performed at 37 °C for 1 h and the reaction stopped by heating at 70 °C for 10 min. A blank control, which contained all the RT-PCR reagents except the viral RNA template, was included with each assay.

### 2.8 Multiplex RT-PCR assay

Viral cDNA (10 μL) was added to buffer a containing 2.5 mM MgCl_2_, 0.3 mM dNTPs, 2.5 U Taq DNA polymerase (Promega, Sydney, Australia), and Taq polymerase buffer (Promega, Sydney, Australia) in a total volume of 25 μL. The reaction mixture contained HEV71-specific primers (20–40 mM), CVA16-specific primers (20 mM) and pan-enterovirus primers F1 and R1 (10–20 mM). Cycling conditions included denaturation at 94 °C for 5 min, followed by 35 cycles of 95 °C for 30 s, 57 °C for 30 s, and 72 °C for 1 min. This cycling was followed by a final extension at 72 °C for 5 min. PCR products were resolved by electrophoresis in 3% agarose gels in TAE buffer and visualised in a gel documentation system (EC3™ Bioimaging system, UVP, Melbourne, Australia). Positive and negative controls were included in each run. A mixture of HEV71 and CVA16 cDNA templates was used as a positive control, whereas nuclease-free water was used as a negative control. Physical separation of sequential steps of PCR was applied in order to prevent DNA cross-contamination between samples. This includes a dedicated bench for sample preparation, a separate UV-treated bench and room for the master mix preparation, and a separate bench and room for the detection of the PCR products. In addition, preparation and pipetting of all solutions were performed using nuclease free, aerosol resistant, sterile pipette tips and tubes.

### 2.9 Specificity of the multiplex RT-PCR assay

The specificity of the multiplex RT-PCR assay was examined using the following enterovirus isolates: HEV71 strains belonging to genotype B3, B5, C2, C4, C5, C1 (Cardosa et al., 2003; Herrero et al., 2003; McMinn et al., 2001) and CVA16 strains (Chua, 2007; Perera et al., 2007; Tu et al., 2007). Other enterovirus strains and other viruses, including Japanese encephalitis virus, dengue virus, rotavirus and herpes simplex virus types 1 and 2, previously isolated and identified at PI-HCMC, were also used for specificity testing (Table 2). Virus was propagated in cell culture until the titre reached approximately 10^6^–10^7^ TCID_50_/mL prior to RNA or DNA extraction.

### 2.10 Sensitivity of single and multiplex RT-PCR assays

Serial dilution of full-length plasmid cDNA clones of HEV71 (Chua et al., 2008), CVA16 and ECHO7 (Chua, 2007) were used to determine the detection limit of the single and multiplex RT-PCR assays used in this study.

## 3 Results

### 3.1 Development and optimisation of multiplex RT-PCR assay

**Fig. 1:**
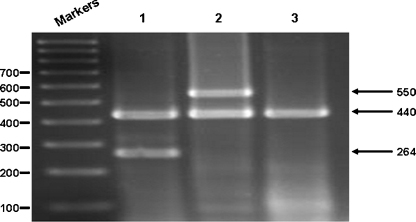
Simultaneous detection of enterovirus 5′UTR and of HEV71 and CVA16 VP1 targets by multiplex-RT-PCR assay at the optimised concentration determined for each of the three primer pairs. Arrows show the CVA16-specific RT-PCR amplicon (550 bp), pan-enterovirus amplicon (440 bp) and the HEV71 amplicon (264 bp). Molecular weight markers are a 100 bp DNA ladder (GeneWorks). Lane 1 – RT-PCR reaction from the HEV71 template; Lane 2 – RT-PCR reaction from a CVA16 template; Lane 3 – RT-PCR reaction from an ECHO7 template.

The multiplex RT-PCR assay required careful optimisation of several parameters, including the MgCl_2_ concentration (final concentration 2.5 mM) and Taq DNA polymerase quantity (final quantity 2.5 U) (data not shown). Most importantly, primer concentrations were titrated to optimise the amplification of all three targets simultaneously in a single multiplex assay. Initial experiments used equal concentrations (20 mM) of the three primer pairs and resulted in variable amplicon band intensities (data not shown). In particular, the HEV71-specific amplicon was present in very low concentration. Optimisation of the three primer pair concentrations in the multiplex assay resulted in concentrations of 40 mM, 20 mM and 10 mM for the HEV71-specific, the CVA16-specific and pan-enterovirus primer pairs, respectively, being chosen for the multiplex assay. These primer concentrations resulted in similar amplicon concentrations being observed upon visualisation of the PCR products after agarose gel electrophoresis (Fig. 1).

### 3.2 Specificity of multiplex RT-PCR assay

The specificity of the multiplex RT- PCR assay was determined by analysing RNA derived from 77 enterovirus strains: 27 strains of HEV71, 15 strains of CVA16 and 35 other enterovirus strains (Table 2). All enteroviruses investigated resulted in the amplification of the 440 bp pan-enterovirus amplicon; all HEV71 strains generated the 264 bp HEV71-specific amplicon plus the 440 bp pan-enterovirus amplicon; all CVA16 strains generated the 550 bp CVA16-specific amplicon plus the 440 bp pan-enterovirus amplicon. Finally, the assay was performed using Japanese encephalitis virus, dengue virus types 1–4 and rotavirus RNA templates and herpes simplex virus types 1 and 2 DNA templates. No observable PCR products were amplified from these templates.

### 3.3 Sensitivity of single and multiplex RT-PCR assays

The sensitivity of the multiplex RT-PCR assay was compared with the single target assays by serial dilution of HEV71, CVA16 and ECHO7 full-length cDNA clones. The sensitivity of the HEV71-specific assay was 10-fold lower (10^3^ copies) in the multiplex PCR assay than in the individual single target assay (10^2^ copies), whereas the sensitivity of the pan-enterovirus and CVA16 assays were identical in both single target and multiplex assays (10^2^ copies).

### 3.4 Validation of multiplex RT-PCR assay for use with clinical specimens

**Fig. 2:**
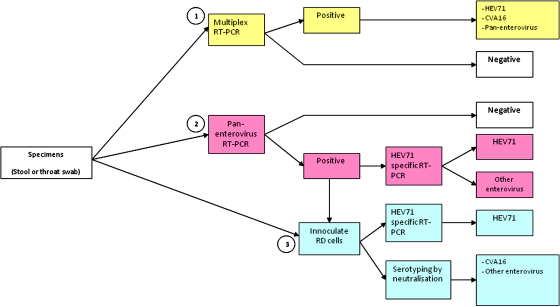
Flowchart showing the procedures used for detection and identification of enterovirus strains derived from clinical specimens and used in the comparison with the multiplex RT-PCR assay.

The ability of the multiplex RT-PCR assay to detect HEV71, CVA16 and other enteroviruses directly from clinical specimens was compared with the methods used currently in our laboratory; a flowchart of the validation procedures undertaken is presented in Fig. 2. A total of 212 specimens (104 throat swabs, 108 stool samples) obtained from the 108 enrolled children were collected for comparison.

**Fig. 3:**
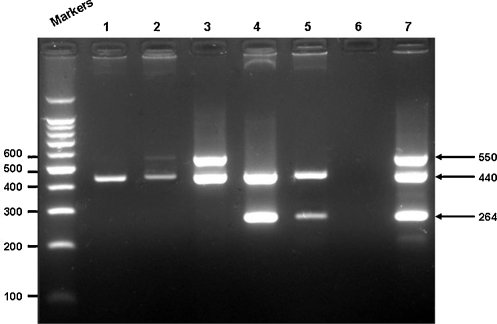
Multiplex RT-PCR data derived from a representative sample of throat swab specimens obtained from the HFMD cases:. Lane 1 – specimen positive for enterovirus (unknown serotype); Lanes 2 and 3 – specimens positive for CVA16; Lanes 4 and 5 – specimens positive for HEV71; Lane 6 – negative control; Lane 7 – positive control (a mixture of infected HEV71- and CVA16-infected cell RNA templates). Pan-enterovirus RT-PCR amplicon size – 440 bp; HEV71-specific RT-PCR amplicon size – 264 bp; CVA16-specific RT-PCR amplicon – 550 bp. Molecular weight markers are the 100 bp DNA ladder (Promega).

RNA extracted from the clinical specimens was used as a template for the multiplex RT-PCR and single pan-enterovirus RT-PCR assays. Specimens positive by pan-enterovirus assay were split, with one aliquot cultured on RD cells and the second aliquot used as a template for the HEV71-specific RT-PCR assay. Cell culture supernatants derived from clinical specimens demonstrating CPE were subjected to (1) RNA extraction for use as templates in the HEV71-specific RT-PCR assay, and, (2) virus neutralisation assays using CVA16- and several echovirus and coxsackievirus B (CVB)-specific antisera. Results of the comparison between the multiplex RT-PCR assay and the single RT-PCR and cell culture-based virus identification methodologies are shown in Table 3 and in Fig. 3.

The multiplex RT-PCR assay was found to be equivalent in both sensitivity and specificity to a diagnostic algorithm that included (1) screening with the single pan-enterovirus RT-PCR, (2) virus isolation and neutralisation assay to identify CVA16 and other enterovirus serotypes, and, (3) single target HEV71-specific RT-PCR assay, in the viral diagnosis of children admitted to hospital with HFMD (Table 3). Of a total of 212 specimens tested from 108 enrolled children, 74 (35%) were found to be positive for HEV71 and 5 (2.4%) positive for CVA16 by both diagnostic algorithms. Fifty (46.3%) HEV71 and four (3.7%) CVA16 infections were identified from the 108 enrolled HFMD cases. Of the fifty the HEV71-infected HFMD patients, virus was detected in both the stool and throat swab specimens of 24 (32.4%) patients, in only the stool of 24 (32.4%) patients and in only the throat swabs of two (2.7%) patients. In the four CVA16-infected HFMD patients, virus was identified in the stool of one (20%) patient and in both the stool and throat swabs of three (60%) patients. Other enteroviruses serotypes identified by neutralisation assay were ECHO25, ECHO4, ECHO6, ECHO7, ECHO14, and one CVB serotype, which were found in 8 (7.4%), 5 (4.6%), 2 (1.9%), 1 (0.9%), 1 (0.9%), and 1 (0.9%) of 108 enrolled HFMD cases, respectively. Ten (9.3%) enterovirus isolates were untypable using the neutralisation antisera available to us. Of the 28 other enterovirus- positive HFMD cases, virus was isolated from both stool and throat swabs in 13 cases, from only stool specimens in 13 cases and from only throat swabs in 2 cases.

HEV71 isolates identified by a single HEV71-specific RT-PCR, and CVA16 and other enterovirus isolates identified by neutralisation test were also subjected to the multiplex RT-PCR for confirmation; no discrepancies were observed between the results obtained by any of these methods (data not shown). Furthermore, the multiplex RT-PCR assay offers the distinct advantage of providing a rapid turnaround time (6–8 h) in comparison with 3–4 weeks for cell culture-based enterovirus identification methods.

## 4 Discussion

Due to a significant increase in HEV71-associated neurological disease in the Asia-Pacific region, there is a need to develop rapid, sensitive, specific and cost-effective assays for the detection and differentiation of HEV71 and other enteroviruses in the laboratory diagnosis of HFMD. Early diagnosis from clinical specimens allows close monitoring and surveillance of HEV71 activity in the population. In this study, the development of a multiplex RT-PCR assay to allow the simultaneous detection of HEV71, CVA16 and other enteroviruses directly from clinical specimens is reported. This assay used smaller quantities of reagents and had a shorter time to completion than cell culture isolation and single RT-PCR assays. Thus, in comparison with the standard procedures of virus isolation and RT-PCR or cell culture-based identification, this assay is more cost-effective and rapid, rendering it more useful for clinical diagnosis and epidemiological surveillance. The identification of HEV71, CVA16 and other enteroviruses by multiplex-RT-PCR required only 6–8 h from specimen receipt to completion.

The primers specific for the HEV71, CVA16 and pan-enterovirus assays were designed to have similar melting temperature and to produce amplicons of different sizes, in order to be compatible in a multiplex assay and to be detected readily by agarose gel electrophoresis, respectively; optimisation of the three primer pair concentrations allowed similar quantities of the amplified products to be detected in the same reaction. As the VP1 gene is the most informative region for molecular epidemiology and evolutionary studies of enteroviruses (Cardosa et al., 2003; McMinn, 2002) we designed primers for the specific identification of HEV71 and CVA16 based on the VP1 gene. Other enteroviruses (i.e. non-HEV71 and non-CVA16), however, could not be serotyped individually by this multiplex PCR assay because the enterovirus 5′UTR is highly conserved. Currently, molecular serotyping of enteroviruses is based on DNA sequencing of the VP1 gene (Oberste et al., 1999). A recent study has reported the use of a RT-PCR assay targeting VP1 for the direct identification of enteroviruses in clinical specimens (Leitch et al., 2009). In future studies, the primers reported in Leitch et al. (2009) could be incorporated into the multiplex RT-PCR assay to allow the identification of other (i.e. non-HEV71 and non-CVA16) enterovirus serotypes associated with HFMD in clinical samples. In this study, other enterovirus serotypes identified by virus neutralisation assays were echovirus and CVB groups, which collectively accounted for 26% of enrolled HFMD cases. With the exception of ECHO14, the echovirus serotypes identified and CVB group viruses, including CVB3, CVB4 and CVB5, have been previously associated with cases of HFMD (Chan et al., 2003; Hooi et al., 2002; Russo et al., 2006). Echoviruses and CVB were the sole viruses identified in specimens collected from 18 HFMD patients, with no other HEV-A viruses identified; the specific serotype was not identified in ten HFMD patients from whom an enterovirus was identified. Thus, it is reasonable to conclude that these CVB and echovirus strains were the causative agents of HFMD in these cases. The results demonstrated a diversity of enterovirus serotypes associated with HFMD cases in Vietnam. Interestingly, other HEV-A viruses, particularly CVA6 and CVA10, have recently emerged as common causes of HFMD outbreaks in other countries, including Singapore and Finland (Blomqvist et al., 2010; Ang et al., 2009). These data emphasise the importance of epidemiological surveillance for monitoring of enterovirus activity in Vietnam.

Similar to that reported previously (Henegariu et al., 1997), the relative concentrations of the primer sets were found to be the most critical factor in optimisation of the multiplex RT-PCR assay. It was necessary to titrate primer concentrations and different concentrations of each primer pair were required to achieve the simultaneous amplification of all three targets. A problem encountered frequently in the development of multiplex PCR assays is a reduction in sensitivity due to competition for reagents when multiple templates are amplified in a single reaction. In this study, the sensitivity of multiplex RT-PCR for detecting HEV71 was reduced ten-fold compared to single target RT-PCR. However, no reduction in the sensitivity of multiplex RT-PCR was observed in the detection of CVA16 and ECHO7 compared with single target RT-PCR assays. Although the sensitivity of the multiplex-PCR was moderately low, with a detection limit of 10^2^ copies, it showed a similar sensitivity to the single pan-enterovirus RT-PCR and HEV71-specific PCR assays in the detection of enteroviruses in clinical specimens (Table 3). This could be due to these clinical specimens containing viral loads higher than the detection limit of the multiplex-PCR. The sensitivity for direct detection of HEV71, CVA16 and other enteroviruses (non-HEV71 and non-CVA16) in specimens obtained from HFMD patients was also higher than that reported in our previous study (Tu et al., 2007). An enterovirus was identified in 82 (76%) of the 108 HFMD patients enrolled in this study: 50 (46.3%) were identified as HEV71, 4 (3.7%) were identified as CVA16 and 28 (26%) were identified as other enteroviruses, whereas in the previous study, 411 (54%) of the 764 HFMD patients were positive for enterovirus infections: 173 (23%) of HEV71, 214 (28%) of CVA16 and 24 (3%) of other enteroviruses. In future studies, the sensitivity of the multiplex RT-PCR could be improved by the use of a real-time PCR platform. Xiao et al. (2009) reported recently that the rate of HEV71 detection in a multiplex real-time RT-PCR assay was 50% in stools and 48.8% in throat swabs and the rate of CVA16 detection was 16.4% in stools and 7% in throat swabs. In this study, HEV71 was detected in 44.4% of stools and in 25% of throat swabs and CVA16 was detected in 3.7% of stools and in 1% of throat swabs. Thus, the multiplex real-time RT-PCR appears to be more sensitive than the multiplex RT-PCR. However, the differences between the two studies could be due to different sample collection and preparation methods. Nevertheless, the sensitivity of our multiplex RT-PCR assay will need to be compared with a real-time RT-PCR assay in a future study.

In conclusion, this study demonstrates that a multiplex RT-PCR assay can be used as a rapid and cost-effective diagnostic tool to detect the presence of HEV71, CVA16 and other enteroviruses in clinical samples derived from HFMD cases. This multiplex RT-PCR is also a potentially useful tool to investigate the molecular epidemiology of HFMD caused by HEV71 and CVA16. Finally, the developed assay is robust and readily adaptable to clinical and public health laboratories in resource limited settings similar to Vietnam.

## Figures and Tables

**Table 1:** Oligonucleotide primers used for the detection of human enteroviruses, human enterovirus 71 and coxsackievirus A16 in single target and multiplex RT-PCR assays.

Virus (PCR target)	Oligonucleotide	Target region	Sequence (5′-3′)^a^	PCR product
Pan-enterovirus (5′UTR)	F1	5′UTR (160–180)	5′- CAAGCACTTCTGTTTCCCCGG-3′	440
Human enterovirus 71 (VP1 gene)	F2	5′UTR (599–580)	5′- ATTGTCACCATAAGCAGCCA -3′	
	EV71–VP1F2	VP1 (2465–2484)	5′- GARAGYTCTATAGGRGAYAG-3′	264
	MAS02A	VP1 (2728–2709)	5′- AGAGGGAGRTCTATCTCYCC-3′	
Coxsackievirus A16 (VP1 gene)	CA16–VP1F	VP1 (2647–2666)	5′- AGGGTAATGGARTGTGGTGAYT-3′	550
	CA16–VP1R	VP1 (3200–3179)	5′- TGTGTGTTGAACCATCACTC-3′	

a R: (A or G), Y: (C or T).

**Table 2:** Specificity testing of the multiplex RT-PCR assay.

Enterovirus serotypes	Species	Total isolates	Positive by multiplex RT-PCR		
			HEV71	CVA16	Pan-enterovirus (5′UTR)
Human enterovirus 71	A	27	27	0	27
Coxsackievirus A16	A	15	0	15	15
Other enteroviruses					
CVA4	A	2	0	0	2
CVA6	A	3	0	0	3
CVA10	A	3	0	0	3
CVB1	B	4	0	0	4
CVB2	B	2	0	0	2
CVB4	B	2	0	0	2
ECHO 6	B	4	0	0	4
ECHO 9	B	3	0	0	3
ECHO 11	B	2	0	0	2
ECHO 19	B	3	0	0	3
ECHO 25	B	4	0	0	4
PV1	C	1	0	0	1
PV2	C	1	0	0	1
PV3	C	1	0	0	1
Other viruses					
Japanese encephalitis virus		1	0	0	0
Rotavirus	A	1	0	0	0
Dengue virus	1,2,3,4	4	0	0	0
Herpes simplex virus	1,2	2	0	0	0
Total		85	27	15	77

CVA, coxsackievirus A; CVB, coxsackievirus B; ECHO, echovirus; PV: sabin poliovirus.

**Table 3:** Comparison of the detection of enteroviruses, human enterovirus 71 and coxsackievirus A16 in stool and throat specimens by multiplex RT-PCR or by a single pan-enterovirus RT-PCR followed by virus isolation and serotype identification by RT-PCR or neutralisation test.

Number tested	Pan-enterovirus RT-PCR, and virus isolation and serotype identification				Multiplex PCR		
	Pan-enterovirus RT-PCR (5′UTR)	HEV71-specific RT-PCR	CVA16- neutralisation	Other enterovirus-neutralisation	Enteroviruses	HEV71	CVA16
Clinical specimens							
108 stool specimens	78	48	4	26	78	48	4
104 throat swabs	42	26	1	15	42	26	1
Clinical cases							
108 cases	82^a^	50^b^	4^c^	28^d^	82^a^	50^b^	4^c^

a 38 cases positive in both stool and throat swabs (24 cases positive for HEV71, 13 cases positive for other enteroviruses, 1 case positive for CVA16), 40 cases positive only in stool specimens (24 cases positive for HEV71, 13 cases positive for other enteroviruses, 3 cases positive for CVA16) and 4 cases positive only in throat swabs (2 cases positive for HEV71, 2 cases positive for other enteroviruses).

b 24 cases positive in both stool and throat swabs, 24 cases positive only in stool specimens and 2 cases positive only in throat swabs.

c 1 case positive in both stool and throat swabs, 3 cases positive only in stool specimens.

d 13 cases positive in both stool and throat swabs, 13 cases positive only in stool specimens and 2 cases positive only in throat swabs.
